# Insulin-Like Growth Factor and Epidermal Growth Factor Signaling in Breast Cancer Cell Growth: Focus on Endocrine Resistant Disease

**DOI:** 10.1155/2015/975495

**Published:** 2015-07-15

**Authors:** Kallirroi Voudouri, Aikaterini Berdiaki, Maria Tzardi, George N. Tzanakakis, Dragana Nikitovic

**Affiliations:** ^1^Laboratory of Anatomy-Histology-Embryology, School of Medicine, University of Crete, 71003 Heraklion, Greece; ^2^Laboratory of Pathology, School of Medicine, University of Crete, 71003 Heraklion, Greece

## Abstract

Breast cancer is the most common type of cancer for women worldwide with a lifetime risk amounting to a staggering total of 10%. It is well established that the endogenous synthesis of insulin-like growth factor (IGF) and epidermal growth factor (EGF) polypeptide growth factors are closely correlated to malignant transformation and all the steps of the breast cancer metastatic cascade. Numerous studies have demonstrated that both estrogens and growth factors stimulate the proliferation of steroid-dependent tumor cells, and that the interaction between these signaling pathways occurs at several levels. Importantly, the majority of breast cancer cases are estrogen receptor- (ER-) positive which have a more favorable prognosis and pattern of recurrence with endocrine therapy being the backbone of treatment. Unfortunately, the majority of patients progress to endocrine therapy resistant disease (acquired resistance) whereas a proportion of patients may fail to respond to initial therapy (de novo resistance). The IGF-I and EGF downstream signaling pathways are closely involved in the process of progression to therapy resistant disease. Modifications in the bioavailability of these growth factors contribute critically to disease progression. In the present review therefore, we will discuss in depth how IGF and EGF signaling participate in breast cancer pathogenesis and progression to endocrine resistant disease.

## 1. Introduction

Breast cancer is the most common type of cancer for women worldwide. Its lifetime risk amounts to a staggering total of 10% where approximately 15–20% of all breast cancers are associated with genetic predisposition [1]. It is well established that breast cancer growth is regulated by the endogenous synthesis of polypeptide growth factors [2] and by growth factors produced at distant sites [3]. Both growth factors and steroids can stimulate proliferation of steroid-dependent tumor cells, and interaction between these signaling pathways occurs at several levels. Indeed, breast cancer is categorized into histopathologic subtypes based on estrogen (ER) and progesterone (PR) hormone receptor status and HER2/ErbB2 epidermal growth factor (EGF) receptors' expression levels. Namely, about 75% of all breast cancers are estrogen receptor- (ER-) positive [4]. This type of breast cancer generally has a more favorable prognosis and pattern of recurrence with endocrine therapy being the backbone of treatment. Antiestrogens and aromatase inhibitors can effectively induce tumor responses in a large proportion of these patients. However, the majority of patients progress during endocrine therapy to resistant disease (acquired resistance) and a proportion of patients may fail to respond to initial therapy (de novo resistance) [4]. Importantly, several steroid responses have now been functionally linked to other intracellular signaling pathways, including c-Src or tyrosine kinase receptors [5]. Moreover, endocrine resistant breast cancer has been correlated to the activation of other signaling pathways, including insulin-like growth factor (IGF) and epidermal growth factor (EGF) pathway [4]. Indeed, endocrine resistance is associated with overexpression of IGF and EGF signaling pathway components, including EGFR, HER2, IGF-IR, and c-Src [6]. Dissecting signaling pathways involved in endocrine and targeted therapy resistant disease is critical for developing novel, more efficient strategies.

## 2. Epidermal Growth Factors Family

The significant role of EGF family members and their respective ErbB receptors in breast cancer cell pathogenesis is well established [7]. The EGF family consists of EGF, transforming growth factor-alpha (TGF-*α*), heparin-binding epidermal growth factor (HB-EGF), amphiregulin (AR), epiregulin (EPR), betacellulin (BTC) and neuregulins (NRGs) [8, 9]. All family members are synthesized as membrane-anchored precursors and their ectodomains cleaved to release the soluble form of growth factor, subsequent to a proteolytic process [7, 10].

The ErbB family of receptors structurally related to EGFR, is composed of ErbB1 (also known as EGFR or HER1), ErbB2 (HER2/Neu), ErbB3 (HER3), and ErbB4 (HER4) [8, 11]. Structurally, the ErbB receptors are composed of an extracellular domain rich in cysteine, a single membrane region and a large cytoplasmic domain [7]. All specific ErbB ligands have an EGF-like domain that endows them with high binding specificity [11, 12]. The extracellular domain is the ligand-binding site of ErbB receptors whereas the cytoplasmic domain has tyrosine protein kinase activity. Mechanistically, the binding of ligands to ErbB receptors leads to their dimerization and subsequent tyrosine phosphorylation of specific tyrosine residues within the cytoplasmic tail [11]. These structural modifications enhance docking of effector proteins whose recruitment induces the activation of downstream signal transduction pathways, including Mitogen-Activated Protein Kinase (MAPK) and Phospatidylinositol-3 Kinase (PI-3K) pathways [7, 13, 14].

### 2.1. EGF Signaling in Breast Cancer Cell Proliferation

ErbB receptors are positively correlated with breast cancer cell proliferation [15, 16]. They are activated by the EGF ligands, EGF, a potent mitogen, being the basic ligand of these receptors, particularly in cells overexpressing EGFR [17, 18]. In addition the ErbB receptors can autophosphorylate or be phosphorylated by other kinases [11]. The activated ErbB receptors bind to Grb2 and Sos downstream mediators, resulting in the activation of intracellular signaling pathways such as Ras/Raf, MAPK, and the PI-3K/Akt pathways which are involved in cell growth, apoptosis, invasion, and migration of breast cancer cells [13, 14, 19, 20]. Specifically, the formation of EGFR-Grb7-Ras complex enhances breast cancer cell proliferation [21].

The key role of EGFR in tumor growth is evident from the antiproliferative effects of monoclonal antibodies specific for this receptor [22]. Furthermore, there is evidence that EGFR activator proteins contribute to EGFR-dependent breast cancer cell proliferation. For example, ERp57 protein, a member of the disulfide isomerase family, participates in the activation of EGFR signaling and in the modulation of its internalization leading to enhanced breast cancer cell proliferation [23]. Moreover, the stimulation of proliferation through activation of EGFR promotes degradation of Fhit protein (the product of tumor suppressor gene* FHIT*) [24].

A concentration-dependent effect of EGF on breast cancer cell proliferation has also been proposed [25–27]. Thus, EGF at concentrations that stimulate most other cell lines reduced the growth of MDA-468 breast cancer cells [25]. Additionally Zhang et al. [28] indicated a biphasic effect of EGF on breast cancer cell proliferation and demonstrated that Src functions as a switch of EGF signaling, depending on EGF concentration. Moreover, EGF seems to induce an inhibition of proliferation through the stimulation of an interleukin 6 type cytokine, oncostatin M, in both estrogen receptor positive and negative breast cancer cells [26]. A novel role for EGF has been proposed by Adams et al. [27]. Specifically, it was found that EGF treatments enhanced miR-206 (microRNA-206) levels in MCF-7 cells, which resulted in reduced cell proliferation, enhanced apoptosis, and reduced expression of multiple estrogen-responsive genes [27].

On the other hand, ezrin-radixin-moesin-binding phosphoprotein-50 (EBP50) suppresses breast cancer cell proliferation [29]. Indeed, EBP50 can suppress EGF-induced proliferation of breast cancer cells by inhibiting EGFR phosphorylation and blocking EGFR downstream signaling [30].

## 3. Insulin-Like Growth Factor Family Members

The biological activities of IGF family not only affect the normal development of the organism but have been strongly implicated in tumorigenesis [31, 32]. This important signaling family consists of the IGF ligands (IGF-I and IGF-II),* their cell membrane* receptors (IGF-IR, IGF-IIR, and IR), and a group of IGF-binding proteins (IGFBPs) [33].

Structurally, whereas insulin is composed of two domains denominated A and B, the IGFs are single-chain molecules that maintain the equivalent of the connecting C-peptide of proinsulin between A and B domains [34]. IGFs are reported to play significant role in cancer progression and according to LeRoith et al. [33] high levels of circulating IGF-I* have been indicated* to constitute a risk factor for the development of breast, prostate, colon, and lung cancer. However, further clinical studies are needed to clarify these first indications.

Importantly, the expression of IGF-I is predictive of breast cancer progression, prognosis, and outcome [32]. The antiapoptotic and mitogenic actions of IGF-I are mediated by its receptor IGF-IR [33, 35]. The IGF-IR activation and overexpression have been implicated in many cellular processes, including cell migration, proliferation, and attenuation of cell survival and are related to the malignant phenotype [31, 36, 37]. IGF-IR is a heterotetrameric transmembrane glycoprotein. Structurally, it forms a *α*_2_ *β*_2_ structure, the *β* subunits endowed with intrinsic tyrosine kinase activity, whereas the *α* subunits are the ligand-binding sites [38, 39]. Binding of ligands to the receptor results in its conformational change and a subsequent autophosphorylation of tyrosine residues 1131, 1135, and 1136 in the kinase domain, juxtamembrane tyrosines, and C-terminal serines [40].

IGF-binding proteins and their respective concentrations regulate the bioavailability of the IGFs and IGF-induced proliferation through IGF/IGFBP complex formation [41–43]. Six high-affinity IGFBPs have been identified to date. Specifically, IGFBP-1 to IGFBP-4 have similar affinities for IGF-I and IGF-II, whereas IGFBP-5 and IGFBP-6 bind IGF-II with higher affinity [44, 45]. All IGFBPs (six proteins) are expressed in mammary tumors [46, 47]. Specifically, IGFBP-4 and IGFBP-5 are expressed in primary breast cancer [46, 47]. Thus, IGFBP-3 provides most of the IGF-binding capacity of serum and greatly prolongs the circulating half-life of the IGFs [48]. Generally, IGFBPs modulate the interactions between IGF ligands and cell-surface receptors [48].

### 3.1. IGF Signaling in Breast Cancer Cell Proliferation

IGF family members (including IGF-I, IGF-II, and their receptors) are overexpressed in breast cancer tumor cells and were shown to promote these cells' survival and growth through various signaling pathways [32]. The principal transduction routes of the IGF signaling are MAPK and PI-3K pathways [49–51]. It is noteworthy that the MAPK and PI-3K/Akt are key mediators of cell proliferation [52–54]. Also, recent data have shown that ERK (MAPK44/42) plays an important role in the resistance of MCF-7 cells to cell apoptosis, which shows the importance of ERK signaling in the extended survival of breast cancer cells [54]. On the other hand, there is evidence that c-Jun N-terminal kinase (JNK) signaling negatively regulates IGF-I induced breast cancer cell proliferation [55].

The majority of IGF-I cellular actions are mediated by the key signaling component of the IGF-I system, the IGF-IR [33]. The activation of IGF-IR by IGF-I results in its autophosphorylation at tyrosine residues [33, 56]. Consecutively, the activated IGF-IR directly phosphorylates other substrates such as IRS-1, IRS-2, and IRS-4 [57]. Upon activation IRS-1 becomes a “docking” protein for other molecules, exhibiting binding sites for SH2 domain-containing proteins [58]. As a consequence, after IRS-1 phosphorylation, many downstream signaling pathways associated with mitogenesis, such as PI-3K [59] and MAPK cascade [60, 61], are activated. Other substrates which are phosphorylated by IGF-IR are src-homology 2/collagen-*α* proteins (Shc) [62], growth factor receptor-binding protein 10 [63], focal adhesion kinase (FAK) [64], and carboxyl-terminal src kinase (CSK) [65].

The importance of IGF signaling in breast cancer is highlighted by reports showing that the IGF-I induced proliferation of MCF-7 breast cancer cells is attenuated with the PI-3K inhibitor LY294002 and the antiestrogen ICI 182780 [66]. In addition, Ahmad et al. [49] suggested that Akt is a downstream mediator of estrogen- and IGF-I-dependent proliferation in hormone-responsive MCF-7 breast carcinoma cells. IGF-I also upregulates Cyr61, a family member of CCN family proteins with many roles in cancer progression, which is characterized by various homologous domains, including the IGF-binding protein domain, through activation of the PI-3K/Akt pathway [67]. This increase in Cyr61 leads to stimulation of breast cancer cell growth and invasion [67]. Furthermore, the inhibition of MAPK or Akt pathways prior to IGF-I stimulation prevents the expression of specific tumor suppressor miRNAs. In a novel report, [51] suggest that IGF-I signaling regulates the expression of specific miRNAs in the estrogen receptor positive MCF-7 breast cancer cell line and indicate kinase signaling as a modulator of expression for a small subset of microRNAs.

Burtrum et al. [68] show that the inhibition of IGF-I signaling, via IGF-IR blockade (generation of specific monoclonal antibody), inhibits the activation of downstream MAPK and Akt signaling pathways. As a result, the proliferative potential effect of IGF-I and IGF-II was reduced. Moreover, IGF-IR deficient mice show a reduced rate of tumor growth and cell migration [69], indicating the central role of IGF-IR in breast cancer cell proliferation. Additionally, the IGF-IR suppression increases apoptosis through p38MAPK phosphorylation in MCF-7 cells [70]. Preclinical findings however have not been translated to date in effective treatment strategies [71]. The function of tumor suppressor genes influences the IGF-IR signals and their downstream proliferative effects on breast cancer cells [48]. Transcription of IGF-IR gene is negatively regulated by tumor suppressors, including the Breast Cancer Gene-1 (BRCA1), p53, and Wilms' tumor protein-1 (WT1) [72, 73]. The role of BRCA1 and BRCA2 in breast cancer progression and prognosis is well documented [74]. BRCA1 inhibits IGF-I action, so* BRCA1* deficiency also leads to increased expression of several IGF-I signaling pathway components in multiple experimental systems, including mice, mammary tumors, and cultured human cells [75, 76]. As a result, mutation or deficiency of* BRCA1* leads to stimulated IGF-I activity and, consequently, increased cell proliferation. Apart from this, the loss of p53 tumor suppressor gene has also been demonstrated to increase IGF-IR expression [77, 78]. This mechanism however does not involve direct DNA binding to IGF-IR promoter sequences [73]. Another example is loss of function of tumor suppressor gene* PTEN* which encodes a phosphatase that attenuates signals originating at tyrosine kinase receptors such as IGF-IR [32, 48].

### 3.2. Insulin-Like Growth Factor and Epidermal Growth Factor Signaling in Endocrine and Targeted Therapy Resistant Breast Cancer Proliferation

It is well established that both steroids and growth factors stimulate proliferation of steroid-dependent tumor cells and that the interaction between these signaling pathways occurs at several levels [6, 49]. The steroid ligands are transferred to the nucleus where they bind steroid receptors, which are classified as ligand-activated transcription factors, to activate target gene transcription and cell growth. Several steroid responses have now been functionally linked to other intracellular signaling pathways, including tyrosine kinase receptors or c-Src. Steroids such as 17 beta-estradiol (E2) via binding to cytoplasmic or membrane-associated receptors were also shown to rapidly activate intracellular signaling cascades including ERK, PI-3K, and STATs [79]. These E2-stimulated phosphorylation-mediated cascades can then contribute to altered tumor cell function [80]. ER-*α* targeted therapy is routinely used to treat breast cancer. However, patient responses are limited by resistance to endocrine therapy [81, 82]. The development of resistance to endocrine therapy often results in uncontrollable growth and dissemination of breast cancer [82]. Therefore, identifying mechanisms that drive endocrine resistance is a high clinical priority.

A large body of experimental evidence indicates that oncogenic signaling pathways underlie endocrine resistance, including EGFR, HER2, IGF-IR, c-Src, and ER itself [6]. The crosstalk between estrogen, IGF-I, and EGF signaling pathways and its involvement in endocrine resistance is well documented in breast cancer [83, 84]. Indeed, breast cancer cells resistant to the pure steroidal ER antagonist fulvestrant demonstrate increased activation of EGFR family members and downstream ERK signaling. Moreover, EGFR has been identified as one of main genes conferring estrogen independence to human breast cancer cells [85]. E2 signaling interacts with IGF-I and EGF pathways, at different levels, for example, through the rapid activation of IGF-IR and EGFR receptors and with the consequent induction of MAPK activation in breast cancer cells [86–88]. Thus, E2 and EGF cues are obligatory in the proliferation of ductal epithelial breast cancer cells and they have synergistic effects [5, 80]. Furthermore, the knockout of EGFR (or IGF-IR) abolished the E2-proliferative response in mice, and the inhibition of EGFR activity suppressed the proliferative effect of E2 in breast cancer cells* in vitro* [79, 89]. In addition, the selective inhibitors or knockdown of both receptors diminished E2-induced MAPK activation but also blocked E2-proliferative action [90]. It is proposed that E2 stimulates the expression of IGF and EGF ligands and receptors in rodent tissues and human cell lines [91]. The ER suppression in breast cancer cells prevents both EGF (and E2) stimulation of DNA synthesis [92]. However, the mitogenic effects of EGF are not mediated by ER, but it is suggested that the crosstalk between the estrogen and EGF signaling pathways may occur by other mechanisms [93]. Notably, both EGFR and c-Src stimulated pathways can induce activation of a transcription factor STAT5, which is needed for E2-induced breast cancer cell proliferation, in some cell lines [79]. Growth factor mediated ER activation is a major route through which breast cancer cells exhibit endocrine resistance to antiestrogen therapies [94]. During the genomic action of E2, binding of E2 to ER*α* results in its activation through separation from heat-shock protein 90 (Hsp90). Active ER-E2 dimer binds directly or indirectly to ERE genes (genes containing estrogen response elements) [95]. On the other hand, the nongenomic actions of E2 lead to the rapid activation of independent signaling pathways, such as IGF-IR and EGFR, depending on cell type [96]. In MCF-7 cells, estrogen potentiates the effect of IGF-I on IGF-IR signaling in the promotion of cell proliferation and protection against apoptosis [97]. Specifically, ER*α* binds to the IGF-IR and activates downstream signaling pathways [98]. Moreover, Yu et al. [99] recently reported the IGF-I induced association of IGF-IR and ER*α*. Furthermore, ER*α* regulates the IGF-I signaling pathways through phosphorylation of ERK1/2 and Akt where the interaction of ER-IGF-IR potentiates breast cancer cell growth [99]. Selective inhibitors or knockdown of IGF-IR (and EGFR) decreased E2-induced MAPK activation and blocked E2 mitogenic effect, confirming crosstalk signaling between IGF-IR, E2, and EGFR [90]. Moreover, E2 was found to utilize a signaling pathway which involves the interactions between ER*α*, IGF-IR, matrix metalloproteinases, and EGF-R to activate MAPK phosphorylation [88]. A consecutive study indicated that the transcriptional activity of ligand free ER is sufficient to complement the mitogenic action of IGF-IR-induced PI-3K/Akt activation [100]. On the other hand, Amin et al. [101] suggested that proliferation of MCF-7 breast cancer cells is suppressed by IGF-I-activated JNK MAPK pathway, through the induction of the SHP1 phosphatase expression.

**Figure 1:**
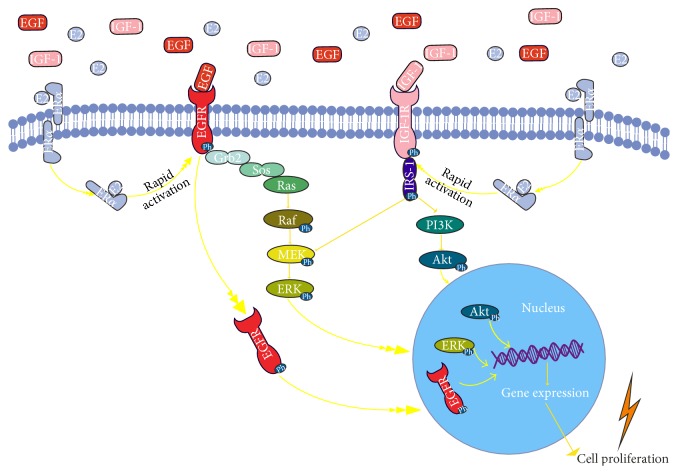
Schematic representation of IGF/EGF signaling, crosstalk with ERs. IGF-I binding to IGF-IR results in its activation and the consequent activation of PI-3K and ERK mediators. EGF-induced activation of EGFR activates the ERK and may lead to gene expression of proliferative genes via internalization of activated EGFR to nucleus. E2-ER*α* binding leads to rapid activation of EGFR and/or IGF-IR. The activation of IGF/EGF signaling pathways stimulates cell proliferation.

Approximately, 15% of breast tumors are classified as triple-negative breast cancers (TNBC), a term that denotes their lack of estrogen receptor and progesterone receptor and nonamplification of the HER2 [102]. Therefore, TNBC patients cannot be treated with endocrine therapy or targeted therapies due to lack of related receptors which results in the poor prognosis. Characteristically, these patients overexpress the EGFR and IGFBP-3 proteins [103] but are resistant to tyrosine kinase inhibitors (TKIs) and anti-EGFR therapies. Up to date different mechanisms have been suggested for resistance to TKIs including EGFR independence, mutations and alterations in EGFR, and its downstream signaling pathways [104]. Efforts have focused on overcoming targeted therapy resistance. Thus, in TNBC cells a Src Family Kinases (SFKs) influenced EGFR translocation to nucleus has been reported, which in turn enhances breast cancer cell growth [105]. Within the nucleus, EGFR can function as a cotranscription factor to regulate genes involved in tumor progression [106], which has been linked to anti-EGFR therapies resistance [107]. Importantly, EGFR contributes to chemo- and radioresistance by enhancing DNA damage repair [107]. Inhibition of EGFR translocation led to a subsequent accumulation of EGFR on the plasma membrane, which greatly enhanced sensitivity of TNBC cells to anti-EGFR therapy [105]. Therefore, targeting both the nuclear EGFR signaling pathway, through the inhibition of its nuclear transport, and the classical EGFR signaling pathway may prove a viable therapeutic approach. Moreover, recently it has been demonstrated that the scaffolding protein NHERF1 sensitizes EGFR-dependent tumor growth, motility, and invadopodia function to anti-EGFR (gefitinib) treatment in TNBC cells [104]. Inhibition of IGFB-3 signaling through sphingosine kinase-1 sensitizes TNBC cells to EGF receptor blockade [108]. Furthermore, the blocking of annexin A2 (a calcium-dependent phospholipid binding protein, present at the surface of triple-negative breast cancer cells) by a specific antibody suppressed the EGF-induced EGFR tyrosine phosphorylation and internalization. Treatment with this antibody also inhibited the EGFR-dependent PI3K-Akt and Raf-MEK-ERK downstream pathways, resulting in reduced cell proliferation [109]. The main IGF/EGF-related signaling mechanisms in breast cancer disease are schematically depicted in Figure 1.

## 4. Bioavailability of Growth Factors

The cellular microenvironment and modifications of extracellular matrix components (ECM) are closely correlated to malignant transformation and all the steps of the metastatic cascade [110, 111]. Namely, the tumor cells do not exist in isolation but rather subsist in a rich microenvironment provided by resident fibroblasts, endothelial cells, pericytes, leukocytes, and ECM components. Indeed, interactions with the tumor microenvironment or stroma are recognized as one of important “hallmarks of cancer.”

In addition the ECM is characterized as a big “tank” containing various mediators including growth factors and cytokine which are either soluble or bound into structural ECM components [112]. We discuss here the fact that bioavailability of EGF and IGF-I, provided by the tumor microenvironment, modulates phenotypic plasticity, gene expression, and the recurrence rate of certain TNBC tumors. Combinatorial therapy with EGFR and IGF-IR inhibitors prevents disease progression by interrupting paracrine interactions between TNBC tumor cells and their microenvironment.

The ECM turnover and remodeling are extremely active in the tumor microenvironment [111]. The ECM components are modified through the actions of various proteases and glycosidases which results in altered ECM structure and bioavailability of key mediators [113]. Indeed, the proteolytic action of various enzymes modulates the bioavailability of soluble growth factors which via the activation of specific intracellular signaling pathways regulate critical tumor cell functions, such as migration and proliferation. The matrix metalloproteinase's family (MMP) which consists of 24 (known) zinc-dependent endopeptidases has a key role in the ECM reorganization that occurs during cancer progression [114]. Specifically, the MMPs' action in the tumor microenvironment creates space for cancer cell migration and regulates their cell proliferation by proteolytical release and activation of the ECM-stored growth factors [114].

## 5. Therapeutic Approach

Targeting of EGF/IGF signaling pathway components has been characterized as a promising approach for breast cancer treatment. Many research groups have focused on the utilization of natural and synthetic inhibitors as well as specific antibodies for EGF family proteins as a strategy to attenuate breast cancer cell proliferation [115–117]. Gefitinib, a tyrosine kinase inhibitor, has been shown to reduce cell proliferation and tumor growth in breast cancer cell lines or* in vivo* conditions in xenografted animals with different levels of EGFR or HER2 expression [118]. Different combinatorial regimes have been approached; thus, the combination of gefitinib with calcitriol or their synthetic analogs resulted in a greater antiproliferative effect than with either of the agents alone in EGFR and HER2 positive breast cancer cells. These effects were due to downregulation of MAPK signaling pathway, decrease of cells in G2/M phase, and induction of apoptosis mediated by upregulation of BIM and activation of caspase 3 [119]. The utilization of monoclonal antibodies is a well-documented approach. Thus, trastuzumab, pertuzumab, and ado-trastuzumabemtansine, which are given intravenously, are monoclonal antibodies that target the ErbB extracellular domains and are used for the treatment of ErbB2-positive breast cancer [120]. The effects of natural products on EGF signaling have also been investigated. Indeed, HMQ1611, a taspine derivative, with anticancer properties, has been shown to reduce the phosphorylation of EGFR and the activation of its downstream signaling mediators ERK1/2 and Akt [117]. Additionally, extracts of* Livistona chinensis* R seeds, with both anticancer and protein kinase inhibitor activity, can attenuate EGF signaling events mainly through EGFR modification [121]. Another agent, (+)-aeroplysinin-1 (a natural metabolite from a type of marine sponge), abolished the proliferative effect of EGF on breast cancer cells and inhibited the ligand-induced endocytosis of the EGFR* in vitro* [122]. However, even though anti-EGFR or combinatorial treatments have been endorsed they are unfortunately meeting with resistance. Current research is focused on increasing our understanding on the mechanisms of response and the discovery of predictive markers. Due to overlapping and redundancy targeting several pathways simultaneously seem essential [123].

Many research groups have endeavored to inhibit the IGF-I signaling, via IGF-IR blockade [68, 124, 125]. This strategy resulted in antiproliferative effects and conclusion that IGF-IR blockade may provide a number of clinical benefits [68]. Furthermore, the effects of natural products on IGF-induced cell proliferation have also been widely studied [124–126]. Thus, it has been reported that samsum ant venom (SAV) inhibits the IGF-I mediated phosphorylation of ERK and AKT, but not p38MAPK [126]. Moreover, deguelin, a natural product isolated from several plant species, has antitumor activity, targeting IGF-IR signaling pathway via upregulation of IGFBP-3 expression [124]. Nimbolide is a terpenoid lactone derived from* Azadirachta indica* (Neem tree) displaying a variety of biological activities. Nimbolide decreases the proliferation of breast cancer cells by modulating the IGF signaling molecules [125]. Calycosin, a natural phytoestrogen with similar structure to estrogen, successfully induced apoptosis of MCF-7 breast cancer cells. Calycosin tends to inhibit growth by ER*β*-induced inhibition of IGF-IR, along with the selective regulation of MAPK and phosphatidylinositol 3-kinase (PI-3K)/Akt pathways [127]. However, even though IGF-IR appeared to be one of promising new targets and early results from clinical trials that targeted the IGF-IR and showed some evidence of response, larger randomized phase III trials have not shown clear clinical benefit of targeting this pathway in combination with conventional strategies [71, 128]. These findings may partly be explained by the complexity of the IGF/insulin system. Thus, surface composition of the receptors, preferential expressions of IRS adaptor proteins, and expression of respective ligands may affect therapeutic outcomes and disease prognosis [129–131]. Therefore, assessment of above factors may be necessary for identification of patients who would benefit from anti-IGFR therapy.

## 6. Conclusions

The bridging between preclinical studies and useful clinical strategies seems to demand a deeper understanding of these complex pathways. Development of predictive molecular biomarkers and optimal inhibitory approaches of the IGF/EGF systems should yield better clinical strategies. In conclusion, unraveling the interaction between the critical signaling pathways in breast cancer biology including ER*α*, EGFR, and IGF components should provide additional new concepts in designing combination therapies.
